# A Novel Walking Detection and Step Counting Algorithm Using Unconstrained Smartphones

**DOI:** 10.3390/s18010297

**Published:** 2018-01-19

**Authors:** Xiaomin Kang, Baoqi Huang, Guodong Qi

**Affiliations:** College of Computer Science, Inner Mongolia University, Hohhot 010021, China; kangxiaomin@mail.imu.edu.cn (X.K.); qiguodong@mail.imu.edu.cn (G.Q.)

**Keywords:** smartphone, walking detection, step counting

## Abstract

Recently, with the development of artificial intelligence technologies and the popularity of mobile devices, walking detection and step counting have gained much attention since they play an important role in the fields of equipment positioning, saving energy, behavior recognition, etc. In this paper, a novel algorithm is proposed to simultaneously detect walking motion and count steps through unconstrained smartphones in the sense that the smartphone placement is not only arbitrary but also alterable. On account of the periodicity of the walking motion and sensitivity of gyroscopes, the proposed algorithm extracts the frequency domain features from three-dimensional (3D) angular velocities of a smartphone through FFT (fast Fourier transform) and identifies whether its holder is walking or not irrespective of its placement. Furthermore, the corresponding step frequency is recursively updated to evaluate the step count in real time. Extensive experiments are conducted by involving eight subjects and different walking scenarios in a realistic environment. It is shown that the proposed method achieves the precision of 93.76% and recall of 93.65% for walking detection, and its overall performance is significantly better than other well-known methods. Moreover, the accuracy of step counting by the proposed method is 95.74%, and is better than both of the several well-known counterparts and commercial products.

## 1. Introduction

With the rapid development of MEMS (Micro-Electro-Mechanical System) techniques and wireless communications, the smartphone as a research platform is attracting more and more attention in both academia and industries due to its merits in the following aspects: easy to carry, ubiquitous operations, rich sensing abilities, etc. Moreover, with the ample motion sensors (i.e., accelerometer, gyroscope and magnetometer) embedded in commercial off-the-shelf (COTS) smartphones, there have emerged an abundant of motion related applications, including tracking and positioning, activity recognition, health monitoring, augmented reality systems, and so on.

Nowadays, the tracking and positioning service based on GPS (Global Positioning System) has become a killer application on smartphones, but is usually out of service in indoor environments on account of the blockage of GPS signals. As such, a variety of alternative technologies [1,2,3,4,5] has been developed for indoor localization. Specifically, the pedestrian dead reckoning (PDR), originated from the dead reckoning techniques aiding the navigation of aircrafts, submarines and guided missiles, was developed to assist in indoor pedestrian localization by estimating the pedestrian heading, step count and step length through fusing the motion sensors [6,7,8,9]. In addition, in the activity recognition domain, most of the existing studies use accelerations collected from a constrained smartphone, namely that the smartphone is attached to the fixed position of the user’s body, so as to distinguish different activities (i.e., stay, walk, run, go upstairs and downstairs) [10,11,12]. Additionally, walking detection and step counting are helpful to identify the physical conditions of or offer health care for patients [13,14,15,16]. It can be envisioned that knowing whether a user starts or is walking (termed human walking detection), and, meanwhile, counting steps will remarkably benefit all the above applications.

Therefore, various walking detection and step counting methods have been developed based on one or both of the following two physical phenomena: the moment when the same heel of a pedestrian strikes the ground once during each gait cycle results in a sudden rise in accelerations [17,18,19,20,21,22]; the cyclic nature of human walking results in cyclic signals measured by motion sensors [13,23,24,25,26]. However, existing methods rely on the usage of dedicated foot-mounted sensors [27] or constrained smartphones [28], which essentially imposes severe limitations on applying these methods in practice. In addition, the methods based on smartphones suffer from limited accuracy, especially when smartphone is held in an unconstrained manner [29], namely that the smartphone placement is not only arbitrary but also alterable. Therefore, precisely identifying walking motion and counting the resultant steps are still challenging.

Among existing studies, most of them adopt accelerometers that are cheap and equipped in almost every COTS smartphone, and the others adopt gyroscopes that are relatively expensive and were not equipped in low-end smartphones a few years ago. Gyroscopes and accelerometers measure the first-order and second-order features, respectively, indicating that gyroscopes are able to capture more precise motion information than accelerometers. Moreover, since walking motion results in cyclic signals as mentioned above, the frequency domain features are intuitively more suitable than time domain features as far as walking detection is concerned.

This paper deals with walking detection and step counting in a practical scenario in which the placement of a smartphone can be arbitrary and also alterable during walking, and proposes a novel algorithm by employing a gyroscope and extracting frequency domain features of the three-dimensional (3D) angular velocities returned by the gyroscope. To be specific, fast Fourier transform (FFT) is adopted to quickly derive the spectrum of the Euclidean norm of 3D angular velocities; a thorough experimental analysis illustrates distinct characteristics in the spectrum irrespective of the smartphone placement, motivating us to design a thresholding technique to decide whether walking is being performed. On these grounds, walking frequency can also be estimated given the spectrum result, so that the steps can be indirectly counted by multiplying the walking frequency and its duration. Finally, extensive experiments are carried out by inviting eight subjects in different realistic scenarios, and a comparison is made by involving both existing well-known algorithms as well as popular commercial products. It is shown that the walking detection accuracy of the proposed algorithm can be as high as 93.76%, which is the highest among all the candidates; the step counting accuracy of the proposed algorithm is 95.74%, which is best as well. Therefore, it is promising to further improve the performance of PDR and applications in relation to pedestrian tracking and localization by using the proposed method.

The contributions of this paper can be summarized as follows. First, we take advantage of gyroscopes and frequency domain features of walking motion to identify walking and count steps. Second, the experimental spectrum analysis reveals that the frequency domain features produced by unconstrained smartphones display similar characteristics, resulting that the proposed method is robust to different smartphone placement. Third, walking frequency is estimated and smoothed by using the spectrum information obtained by FFT, which simplifies the implementation of counting steps compared to other approaches like peak detection, autocorrelation, etc. Finally, extensive experiments are conducted by evaluating not only the well-known counterparts but also several popular commercial products.

## 2. Background

In general, the existing studies can be categorized into time domain approaches, frequency domain approaches and feature clustering approaches, which will be briefly reviewed in what follows.

The time domain approaches include thresholding [17,18], peak detection [19,20,21,22], zero-crossing counting [26], autocorrelation [13,23,24,25], etc.

- The thresholding approach counts steps by judging whether sensory data satisfy some predefined thresholds that differ according to various devices held by different users at different positions. In [17], the authors used different states (e.g., not walking, possibly starting a step, stand stationary, etc.) and corresponding thresholds to calculate steps. In [18], the authors tied a sensor to the user’s ankle, and, as long as the acceleration exceeds a threshold, the step count will be increased by one accordingly. However, though the thresholding approach is simplest, it is really difficult to select one optimal threshold for all the cases, especially when smartphones are used in an unconstrained manner.
- The peak detection approach estimates steps based on the number of peaks given a sequence of sensory data, and does not rely on predefined thresholds, but suffers from interference peaks due to environmental noises and occasional disturbance. In [19], authors used low-pass filtering to remove interferences. In [20], the authors limited the time interval between two peaks to reduce misjudgment. In [21], the authors apply two filters to reduce jitters in accelerations. In [22], vertical acceleration data are used to infer steps for unconstrained smartphones, but sensor fusion is required to obtain vertical acceleration data.
- Similarly, the zero-crossing counting approach counts steps by detecting the number of zero points in sensory data, which is susceptible to disturbing sensory data and usually needs to filter and smooth original sensory data in advance. In [26], raw gyroscope data are filtered using a 6th order Butterworth filter for noise reduction. Both of the peak detection approach and zero-crossing approach search for the periods inherent in the cyclic nature of walking by using the magnitudes of accelerations or angular velocities, and can achieve better performance with e.g., vertical accelerations, but are degraded if the smartphone is not firmly attached to a human body.
- The autocorrelation approach detects cyclic periods directly in the time domain through evaluating autocorrelation [25], and is able to obtain good performance at relatively low costs in comparison with the frequency domain approaches. In [6,7], the horizontal components of accelerations and vertical component of angular velocities are respectively adopted for evaluating their autocorrelations and display good detection accuracy, but suffer from high computational costs for transforming reference systems.

The frequency domain approaches focus on the frequency content of successive windows of measurements based on short-term Fourier transform (STFT) [30], FFT [31], and continuous/discrete wavelet transforms (CWT/DWT) [30,32,33,34], and can generally achieve high accuracy, but suffer from either resolution issues [34] or computational overheads [35]. In [31], steps are identified by extracting frequency domain features in acceleration data through FFT, and the accuracy of 87.52% was achieved. Additionally, FFT was employed in [36] too smooth acceleration data and then peak detection was used to count steps.

The feature clustering approaches employ machine learning algorithms, e.g., Hidden Markov models (HMMs) [37,38,39], KMeans clustering [40,41], etc., in order to classify activities based on both time domain and frequency domain features extracted from sensory data [14,42], but neither a single feature nor a single learning technique has yet been shown to perform the best [42].

A fair and extensive comparison has been made among various techniques in a practical environment in [29], and shows that the best performing algorithms for walking detection are thresholding based on the standard deviation and signal energy, STFT and autocorrelation, while the overall best step counting algorithms are windowed peak detection, HMM and CWT.

To sum up, even though great efforts have been invested, it is still challenging to detect and count steps with unconstrained smartphones in an accurate and efficient manner. In this paper, we adopt the gyroscope that is becoming more and more popular in COTS smartphones and the efficient FFT method to implement a novel and practical method for simultaneous walking detection and step counting. Due to the advantages of the gyroscope and frequency domain approach, the proposed method relieves the restriction of most existing studies that assume the usage of smartphones in a constrained manner.

## 3. Methodology

The proposed algorithm firstly decides whether walking is performed based on the Fourier spectrum obtained by FFT because the frequency domain features are relatively stable no matter where a smartphone is placed and incur slight changes with regards to different users walking in various situations.

**Figure 1:**
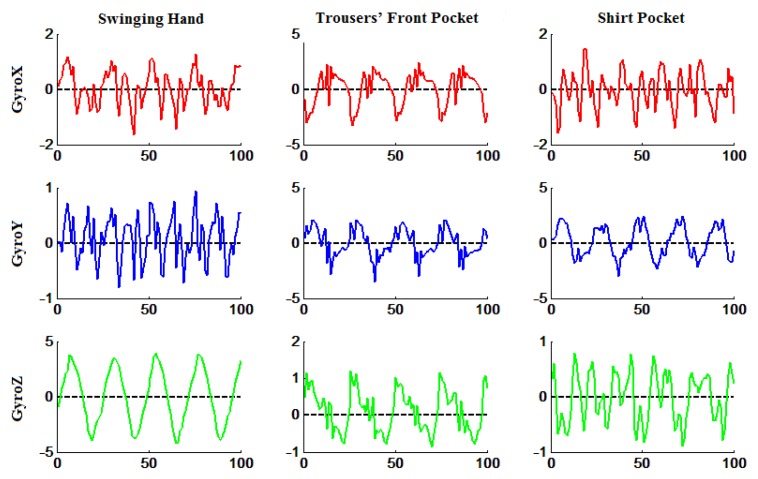
The 3D data derived by the gyroscope of a smartphone placed in three different positions. The horizontal axes denote time.

Differently from most of the existing algorithms that rely on accelerations, the proposed algorithm utilizes angular velocities measured by the gyroscope embedded in COTS smartphones, due to the following aspects: (1) the gyroscope is more sensitive and more accurate than the accelerometer; (2) accelerations suffer from jitters and local minima, whereas angular velocities are smooth and in a good cyclicity regardless of the smartphone placement; and (3) angular velocities normally oscillate around zero during walking, as shown in Figure 1, and are thus more suitable for detection than accelerations.

Additionally, on the basis of spectrum information, the walking frequency can be instantly estimated with respect to every user in different situations, such that the steps can be adaptively counted by multiplying the walking frequency and walking duration. Since it is unnecessary to detect and count specific peaks or zero-crossing points that are severely polluted by noises, relatively accurate step counts are expected.

**Figure 2:**
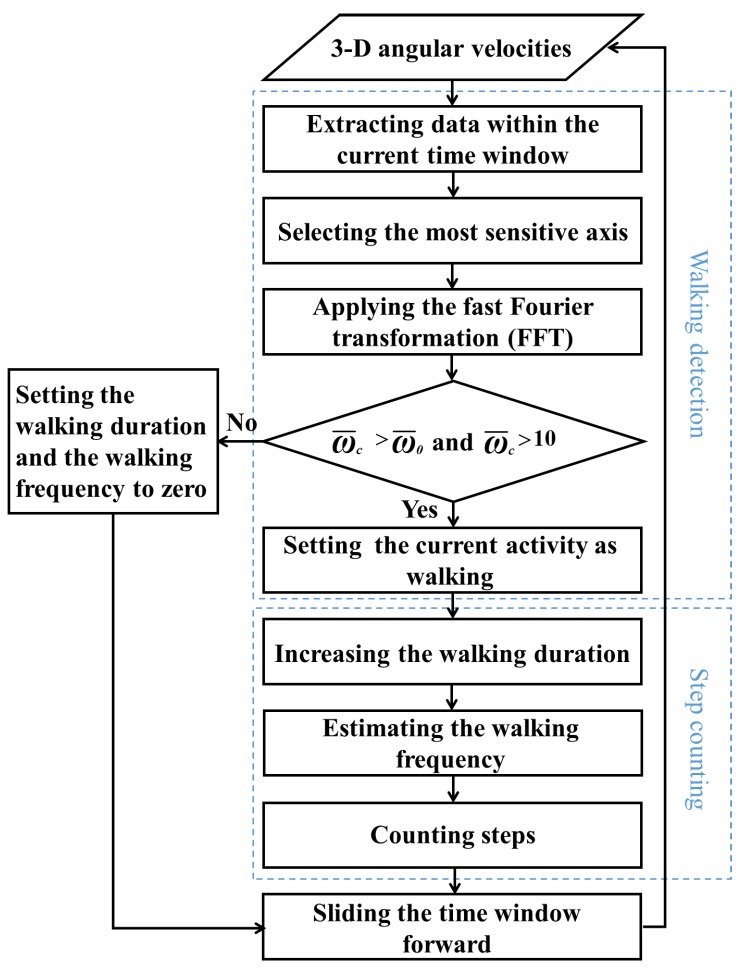
The flow diagram of the walking detection and step counting algorithm.

The flow diagram of the proposed algorithm is illustrated in Figure 2. As can be seen, the algorithm involves two parts, i.e., the walking detection part and the step counting parts, which will be explained in detail in the following two sections.

## 4. The Walking Detection Part

The walking detection part relies on sliding time window, selecting the sensitive axis and spectrum analysis, which will be elaborated in what follows.

### 4.1. Sliding Time Window

In order to continuously detect walking activities, the algorithm is designed on the basis of a sliding time window. As suggested in [43], the typical walking frequency of human ranges from 0.6 Hz to 2 Hz; in other words, the duration of the walking activity approximately ranges between 0.5 s and 1.6 s. Hence, the time window should contain a sequence of data longer than 1.6 s and the sliding step is less than 1.6 s.

Moreover, according to the Shannon Sampling Theory, it is sufficient that the sampling frequency is more than two times the maximum walking frequency. As such, by trading off the energy consumption and minimal sampling frequency, the sampling frequency is set to be 20 Hz.

Since the base-2 FFT algorithm is adopted, the number of instances in each time window is the power of 2, so that it is reasonable to let the time window include 64 instances, with the result that the corresponding duration at the sampling frequency of 20 Hz is 3.15 s and is slightly longer than that of one step in most cases. Ideally, the sliding distance should be equal to the duration of one step, which actually differs with various persons under various situations, and, as such, we let the sliding distance be 1.2 s, which includes 25 instances, which is around the normal duration of one step.

### 4.2. Selecting Sensitive Axis

Supposing that a smartphone holder is asked to perform an identical activity repeatedly, it is true that the 3D data derived by the gyroscope of the smartphone in the device reference frame demonstrate different characteristics according to the position and attitude of the smartphone, as shown in Figure 1, and thus will play different roles in successfully identifying the activity. Therefore, it is of great importance to select the most sensitive axis in the sense that the corresponding data are closely correlated with the activity. An alternative approach is to use the magnitude of the corresponding 3D data instead of the sensitive axis, but inevitably suffers from information loss.

The measurements of the gyroscope incur constant bias, thermo-mechanical white noise, flicker noise or bias stability, temperature effects, and calibration errors (e.g., scale factors, alignments and output linearities). In general, the measurement noises appear to be quite obvious when the measurements are relatively small, and, on the contrary, can be ignored when the measurements are huge. Therefore, it is advisable to select the axis whose data has the maximum magnitude. Moreover, regarding the walking activity, no matter where the smartphone is placed, certain cyclic features are always involved in all the 3D data. Inspired by the above analysis, we come up with the following simple method based on the absolute values of the 3D angular velocities to select the sensitive axis in the proposed algorithm, namely

(1) $$\mathrm{The}\;\mathrm{most}\;\mathrm{sensitive}\;\mathrm{axis}=\max_{a=x,y,z}\sum_{i=1}^{N}\left|\omega_{a}\left(i\right)\right|\;,$$

where $\omega_{a}\left(i\right)$ denotes the angular velocity of the axis *a* with a=x,y,z at time *i* within the current time window, and *N* is the size of the time window and equals 64.

### 4.3. Spectrum Analysis

Based on the above steps, we can determine the sensitive axis of angular velocities, and the corresponding data are fed into the process in this step.

FFT is applied to transform the time domain angular velocities into the following frequency domain data

(2) $$X\left(k\right)=\sum_{n=0}^{N-1}\omega \left(n\right){\left(e^{-j\frac{2\pi }{N}}\right)}^{nk},$$

where k=0,1,…,N−1 and ω(n) is the angular velocity in the most sensitive axis. The frequency of the *n*-th point after transformation, denoted $f_{n}$, can be calculated as follows:

(3) $$f_{n}=\left(n-1\right)\times \frac{f_{s}}{N},$$

where $f_{s}$ is the sampling frequency and equals to 20 Hz.

**Figure 3:**
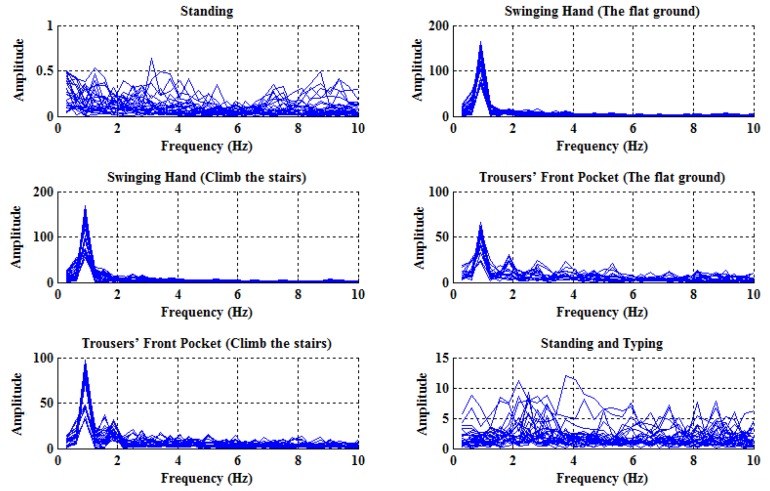
The spectrums of the angular velocities with respect to six different activities.

**Figure 4:**
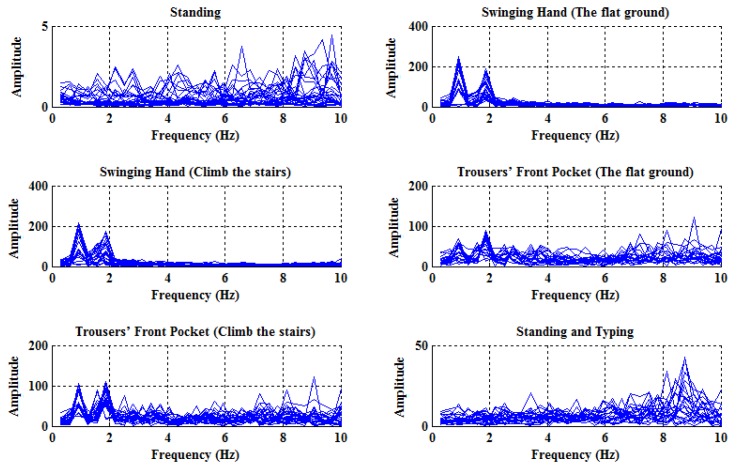
The spectrums of the accelerations with respect to six different activities.

In order to investigate the influences of different activities, the spectrums of the angular velocities with respect to six usual activities are plotted in Figure 3, and, similarly, the spectrums of the accelerations are also plotted in Figure 4 for comparison purposes. Specifically, each subfigure corresponds to one specific activity and contains 24 curves, each of which reflects the spectrum from one of eight subjects and obtained in one sliding time window.

As can be seen from Figure 3 and Figure 4, the spectrums of angular velocities display distinct features in comparison with accelerations, in the sense that in the cases of walking, there exist obvious peaks near 0.9375 Hz, which is close to the normal walking frequency; that is to say, it does make sense to use frequency domain features of angular velocities.

**Figure 5:**
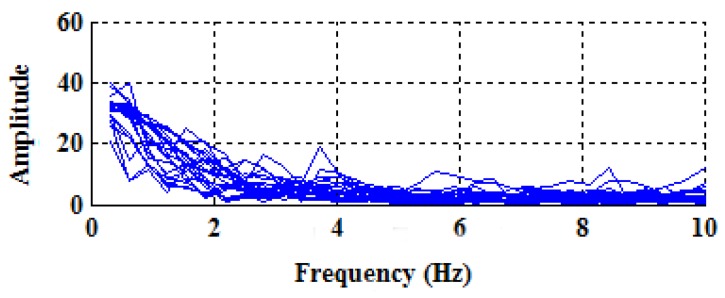
The spectrums of the angular velocities produced by switching among different smartphone placement.

Additionally, the spectrums of the angular velocities produced by switching among different smartphone placement (e.g., picking out the smartphone from the trouser’s front pocket of one subject) are plotted in Figure 5. It can be seen that there is no sudden peak in the vicinity of 0.9375 Hz, implying that it is easy to filter out such kinds of interfering activities from walking.

Inspired by the observation, we propose identifying walking activities by comparing the amplitudes of different frequencies. Specifically, the average amplitude within the typical walking frequency range (i.e., between 0.6 Hz and 2 Hz), denoted by ${\bar{\omega }}_{c}$, and that, between 0 Hz and 0.6 Hz, denoted by ${\bar{\omega }}_{0}$, are evaluated, respectively, and, then, walking is identified if and only if the following condition is satisfied:

(4) $${\bar{\omega }}_{c}>{\bar{\omega }}_{0}.$$

As illustrated in Figure 3, when the holder is operating the smartphone (e.g., standing and typing), the resulting amplitudes are relatively small, reflecting that the smartphone is experiencing some mild motions that do not involve walking; however, in this situation, it often happens that the condition in (4) is satisfied such that incorrect detection results are returned. Therefore, a supplementary condition is imposed by thresholding the average amplitude as follows:

(5) $${\bar{\omega }}_{c}>10,$$

where the lower bound 10 is experimentally determined.

To sum up, if and only if the conditions (4) and (5) are simultaneously satisfied, it is inferred that the smartphone holder is walking.

## 5. The Step Counting Part

As mentioned perviously, the proposed method count the steps by multiplying the walking frequency (denoted $f_{w}$) and the duration of continuous walk (denoted *t*), namely

(6) $$c=t\times f_{w},$$

where *c* denotes the step count.

Consequently, there is no need to count steps by detecting various patterns (e.g., thresholds, peaks, zero-crossing points, etc.), which are vulnerable to noises, with the result that both the computational overheads and step counting accuracy are improved. To do so, the step counting part is composed of three steps, i.e., increasing the walking duration, estimating the walking frequency and counting steps, as illustrated in Figure 4.

In the first step, as long as the walking activity is identified in the current time window, the walking duration is increased by summing 1.2 s, i.e., the size of the sliding step.

In the second step, the walking frequency is estimated on the basis of the spectrums obtained in the walking detection part. In general, during the walk of a smartphone holder, the walking frequency should correspond to the maximum amplitude in the resulting spectrum. However, since the frequencies in the practical spectrums are quantized, directly assigning the frequency with the largest amplitude to the walking frequency is definitely inaccurate and unacceptable. Therefore, the curve fitting technique is adopted to regress the most possible value of the walking frequency. Specifically, the following polynomial fitting function is established

(7) $$A=af^{4}+bf^{3}+cf^{2}+df+e,$$

where *A* is the amplitude, *f* denotes the frequency, and a,b,c,d and *e* are constant coefficients with a≠0.

Since the walking frequency is generally between 0.6 Hz and 2 Hz, then the amplitudes at the frequencies within this range are used to fit (7). As a result, there are five samples whose frequencies can be determined by (3) with the value of *n* varying from 2 to 6. After that, the frequency falling in this range and maximizing the amplitude *A* is returned as the estimate of the walking frequency.

Additionally, the weighted moving average filter is applied to smooth the estimate, namely

(8) $${\bar{f}}_{w}^{i}=\alpha {\bar{f}}_{w}^{i-1}+\left(1-\alpha \right){\hat{f}}_{w}^{i},$$

where ${\bar{f}}_{w}^{i}$ denotes the estimate of the walking frequency after smoothing in the *i*-th time window, ${\hat{f}}_{w}^{i}$ denotes the estimate of the walking frequency in the *i*-th time window, and α denotes the weight and is set to be 0.8.

In the third step, the accumulated step count after the *i*-th time window can be determined using the walking duration *t* and the walking frequency ${\bar{f}}_{w}^{i}$ according to (6).

## 6. Experimental Results

In this section, extensive experiments are conducted and a thorough performance analysis is reported by comparing the proposed method with both existing well-known methods and commercial products.

### 6.1. Setup

**Figure 6:**
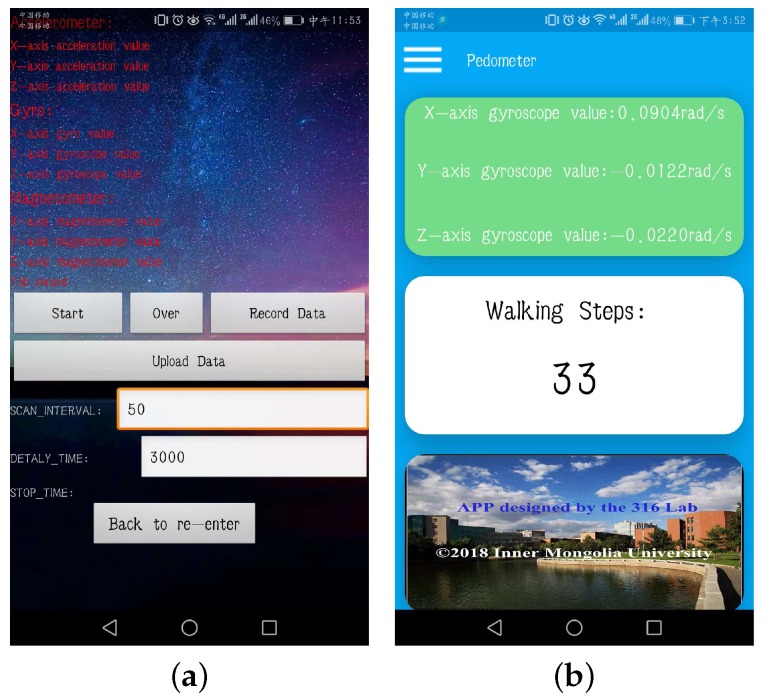
The screenshots of two Android apps. (**a**) sensory data collection; (**b**) step counter using the proposed method.

In the experiments, an app (see Figure 6a) is developed and installed on an Android smartphone (RedMi Note 2, MI, Beijing, China) to collect both acceleration and angular velocity data at the sampling frequency of 20 Hz, and eight subjects, including five males and three females with their ages between 23 and 26, heights between 159 cm and 176 cm, and weights between 54 kg and 80 kg, are invited to continuously perform sequences of predefined daily activities (see Table 1 and Table 2) in the building of the College of Computer Science, Inner Mongolia University. In addition, another app is developed to implement the proposed method as shown in Figure 6b.

In the first scenario, every subject is asked to complete a sequence of different activities listed in Table 1 as usual. Essentially, the sequence of activities corresponds to the real activities performed by these subjects everyday, namely walking from the entrance of the building to the lab in the third floor and meanwhile changing the smartphone placement. This scenario involves frequent switches between different walking activities and non-walking activities, for the purpose of validating the walking detection accuracy of the proposed method.

In the second scenario, every subject is asked to perform different walking activities listed in Table 2 for a long duration, so as to produce a relative large amount of steps and validate the step counting accuracy of the proposed method.

Two well-known walking detection methods belonging to the time domain approaches and frequency domain approaches, respectively, i.e., thresholding based on the standard deviation of accelerations [28] (denoted STD_TH), and STFT based on accelerations [30] (denoted STFT), are realized for comparison because both of them are shown to be the best performing methods for walking detection with an unconstrained smartphone in [29]. Additionally, we implement a copy of the proposed method but using accelerations instead of angular velocities, denoted FFT+ACC, in order to verify the advantage of the proposed method.

Moreover, two well-known step counting methods, i.e., the autocorrelation based method [25] (denoted AC) and the peak detection based method [20] (denoted PD), are realized for performance comparison. In addition, the method reported in [31] (denoted FA) is also realized as a representative of the frequency domain approaches. In addition, three popular commercial products that have been recommended as good applications of pedometer by the online APP market, i.e., Pacer, Spring Rain (denoted SR) and LeDongLi (denoted LDL), are employed here for comparison.

The parameters associated with the proposed and comparing methods are listed in Table 3. All the parameters of the comparing methods are carefully determined based on not only the suggestions in the corresponding papers, but also minor adjustments according to the sensory data collected in the experiments.

### 6.2. Experimental Results of Walking Detection

In order to have a clear knowledge about the performance of the walking detection methods, both precision (denoted *P*) and recall (denoted *R*) are evaluated as below:

(9) $$\begin{array}{ccc} P & = & \frac{\mathrm{TP}}{\mathrm{TP}+\mathrm{FP}}\times 100\%, \end{array}$$

(10) $$\begin{array}{ccc} R & = & \frac{\mathrm{TP}}{\mathrm{TP}+\mathrm{FN}}\times 100\%, \end{array}$$

where TP is the true positive duration of walking, FP is the false positive duration of walking and FN is the false negative duration of walking.

The precision and recall of the performance of the proposed method with respect to different counterparts given different subjects are listed in Table 4. As can be seen, in general, the frequency domain methods outperform the time domain method (i.e., STD_TH), indicating that the frequency domain features are more suitable for walking detection than the time domain features; the proposed method outperforms its copy using accelerations (i.e., FFT+ACC), confirming the advantage of gyroscope over accelerometer in walking detection, and both of them outperform the other frequency domain method (i.e., STFT), verifying the advantage of the frequency domain features based on FFT.

Moreover, it can be observed that different subjects produce quite different detection results. On average, the precisions of the proposed method and its copy using accelerations are around 93% and significantly higher than those of the other two methods. Regarding the STFT method, though its recall is as high as 97.33% on average and is slightly higher than the proposed method, its precision is as low as 73.77%. Evidently, the proposed method is superior to all the other counterparts.

**Figure 7:**
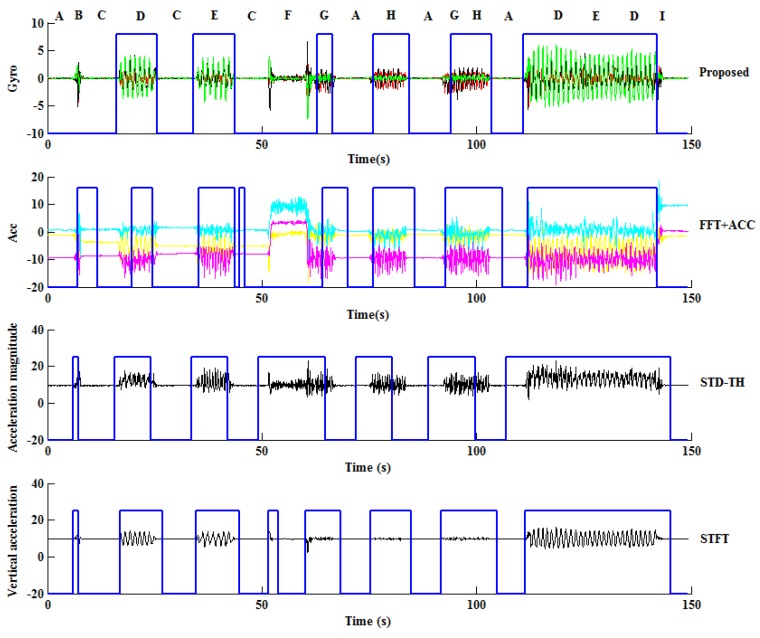
The walking detection results of one subject by using different methods in the first scenario.

In addition, in order to have a clear view, the results of walking detection associated with one subject in the first scenario are depicted in Figure 7. The symbols on the top indicate the activities performed in the corresponding period of time, and the blue solid broken lines reflect the detection results with the upper horizontal line segments being walking and the lower ones being non-walking. In the activity sequence performed, symbols D, E, G and H represent walking activities, and the others represent non-walking activities.

As can be seen, the proposed method and its copy using accelerations appear to be optimal, and both of them outperform the other two methods. Particularly, the proposed method and its copy are able to differ standing and typing action (with symbol F) from walking activities, which cannot be done by the other two methods; in addition, the proposed method denies the other two non-walking activities with symbols B and C as walking, but its copy using acceleration cannot. However, the results are consistent with the overall results in Table 4, and confirm the superiority of the proposed method.

### 6.3. Experimental Results of Step Counting

Define *A* to be the step counting accuracy as follows:

(11) $$A=\left(1-\frac{\left|s_{e}-s_{a}\right|}{s_{a}}\right)\times 100\%,$$

where $s_{e}$ is estimated step count and $s_{a}$ is the actual step count.

The accuracies of different step counting methods and products in the second scenario are listed in Table 5. It can be seen that, even though the proposed method does not always perform best with regard to every considered walking activity, its accuracies are close to the optimal ones and its average accuracy is as high as 95.74%, which is higher than both of the comparing algorithms by at least 3.81% and the commercial products by at least 3.39%. Moreover, the autocorrelation method (denoted AC) and frequency-based method using accelerations (denoted FA) achieve similar performance, which is worse than those of the peak detection method (denoted PA) and the proposed method. Regarding the commercial products, the average accuracies range between 82.94% and 92.35%, and LeDongLi performs best.

By taking into account of walking activities, it can be observed that the accuracies span a wide range between 62.78% and 98.84%, and walking on flat ground often results in good performance compared to climbing upstairs. Regarding the performance of intermittently walking and continuously walking, the difference is significant when the smartphone is held in one’s hand, but trivial when the smartphone is placed in a trouser’s pocket. However, the performance of the proposed method appears to be stable in all the cases, whereas that of the other methods and products experience certain fluctuations, which confirms the robustness of the proposed method.

In summary, the experiments have verified that the proposed method is able to deliver superior performance in both walking detection and step counting under various complicated situations compared to the existing well-known methods and popular commercial products.

## 7. Conclusions

In this paper, we proposed a novel walking detection and step counting method for users with unconstrained smartphones. Differently from most existing studies, the proposed method adopted the gyroscope data and extracted critical walking features in the frequency domain. Moreover, an indirect step counting method was reported by multiplying the walking frequency and the walking duration to mitigate the negative influences of random noises in the time domain. Finally, a thorough experimental analysis was presented by taking into account various complicated but real situations, and showed that the proposed method is able to achieve superior and stable performance in comparison with both existing well-known methods and popular commercial products. This work will not only benefit many existing relevant applications, but also accelerate the emergence of more applications relying on accurate walking detection and step counting. Regarding future works, we would like to apply the proposed method in smartphone oriented localization and navigation.

## 8. Patents

The proposed method is applying for a patent and now has been handed over to the agency.

## Figures and Tables

**Table 1:** The symbols for different daily activities in the first scenario.

Symbol	Daily Activities
A	Standing with the smartphone in the trousers’ front pocket
B	Taking out the smartphone from the trousers’ front pocket
C	Standing and holding the smartphone in the hands
D	Walking on the flat ground with the smartphone in the swinging hand
E	Climbing the stairs with the smartphone in the swinging hand
F	Standing and typing
G	Walking on the flat ground with the smartphone in the trousers’ front pocket
H	Climbing stairs with the smartphone in the trousers’ front pocket
I	Sitting down with the smartphone in the hand

**Table 2:** The symbols for different walking activities in the second scenario.

Symbol	Different Walking Activities
J	Continuously walking on the flat ground with the smartphone in the swinging hand
K	Continuously walking on the flat ground with the smartphone in the trousers’ front pocket
L	Intermittently walking on the flat ground with the smartphone in the swinging hand
M	Intermittently walking on flat ground with the smartphone in the trousers’ front pocket
N	Continuously climbing the stairs with the smartphone in the swinging hand
O	Continuously climbing the stairs with the smartphone in the trousers’ front pocket

**Table 3:** Parameter values used by different methods in the experiments.

Method	Frequency/Time	Window Size (s)	Sliding Distance (s)	Threshold
Proposed	Frequency	3.15	1.2	10
FFT+ACC	Frequency	3.15	1.2	22
STFT	Frequency	3	0.7	20
STD_TH	Time	1.25	1.25	0.74
AC	Time	[1,3.2]	[0.5,1.6]	0.5
PD	Time	1.25	0.5	[1.2g,3g]
FA	Frequency	0.75	0.5	[155,280]

(*g* is the gravity unit.)

**Table 4:** The results of walking detection in the first scenario.

NO.	Proposed		FFT+ACC		STD_TH		STFT	
	P (%)	R (%)	P (%)	R (%)	P (%)	R (%)	P (%)	R (%)
1	90.01	96.84	89.29	91.94	67.80	97.41	73.89	99.11
2	93.24	91.06	94.84	88.65	62.03	88.96	74.67	95.46
3	93.90	96.48	94.83	87.00	60.81	85.12	73.69	97.30
4	95.23	91.24	94.40	83.53	49.31	89.13	68.84	99.84
5	92.74	95.83	93.10	88.91	62.83	74.44	79.40	95.97
6	95.83	89.77	87.65	77.86	45.89	54.83	77.56	93.01
7	94.63	92.61	91.96	86.94	54.81	73.64	65.56	99.01
8	94.51	95.35	93.78	93.01	62.66	88.71	76.55	98.90
Average	93.76	93.65	92.48	87.23	58.27	81.53	73.77	97.33

**Table 5:** The accuracies of different step counting methods and products in the second scenario.

Method.	J	K	L	M	N	O	Average
	A (%)	A (%)	A (%)	A (%)	A (%)	A (%)	A (%)
Proposed	98.55	95.83	96.47	96.76	94.9	91.95	95.74
AC	95.83	88.84	95.55	88.06	78.76	70.2	86.21
PD	95.62	98.43	93.34	94.91	83.46	85.83	91.93
FA	93.49	86.67	86.47	86.15	95.86	69.32	86.33
Pacer	92.42	95.27	84.56	93.58	78.93	91.32	89.35
Spring Run	76.24	89.85	62.78	97.18	79.59	91.98	82.94
LeDongLi	94.35	95.99	90.39	95.91	87.64	89.84	92.35
Average	92.36	92.98	87.08	93.22	85.59	84.35	89.26

## Abbreviations

The following abbreviations are used in this manuscript:

3D	three-dimensional
AC	the autocorrelation based method
COTS	off-the-shelf
CWT/DWT	continuous/discrete wavelet transforms
FFT	fast Fourier transform
FFT+ACC	the proposed method using accelerations
GPS	global positioning system
HMMs	hidden Markov models
LDL	LeDongLi
PD	the peak detection based method
PDR	The pedestrian dead reckoning
SR	spring rain
STD_TH	standard deviation of accelerations
STFT	short-term Fourier transform
